# Exploring the impact of engagement in mental health and substance use research: A scoping review and thematic analysis

**DOI:** 10.1111/hex.13779

**Published:** 2023-06-06

**Authors:** Natasha Y. Sheikhan, Kerry Kuluski, Shelby McKee, Melissa Hiebert, Lisa D. Hawke

**Affiliations:** ^1^ Institute of Health Policy, Management and Evaluation University of Toronto Toronto Ontario Canada; ^2^ Institute for Better Health, Trillium Health Partners; ^3^ Centre for Addiction and Mental Health Toronto Ontario Canada

**Keywords:** lived experience, mental health, patient and public involvement, patient engagement, patient‐oriented research, substance use

## Abstract

**Background:**

There is growing evidence demonstrating the impact of engaging people with lived experience (PWLE) in health research. However, it remains unclear what evidence is available regarding the impact of engagement specific to mental health and substance use research.

**Methods:**

A scoping review of three databases and thematic analysis were conducted. Sixty‐one articles that described the impact of engagement in mental health and substance use research on either individual experiences or the research process were included.

**Results:**

Key topics include (a) the impact of engagement on individual experiences; (b) the impact of engagement on the research process; and (c) facilitators and barriers to impactful engagement. Studies largely focused on the perceived positive impact of engagement on PWLE (e.g., personal and professional growth, empowering and rewarding experience, feeling heard and valued), researchers (e.g., rewarding experience, deeper understanding of research topic, changes to practice), and study participants (e.g., added value, fostered a safe space). Engagement activities were perceived to improve facets of the research process, such as improvements to research quality (e.g., rigour, trustworthiness, relevance to the community), research components (e.g., recruitment), and the research environment (e.g., shifted power dynamics). Facilitators and barriers were mapped onto the lived experience, researcher, team, and institutional levels. Commonly used terminologies for engagement and PWLE were discussed.

**Conclusion:**

Engaging PWLE—from consultation to co‐creation throughout the research cycle—is perceived as having a positive impact on both the research process and individual experiences. Future research is needed to bring consistency to engagement, leverage the facilitators to engagement, and address the barriers, and in turn generate research findings that have value not only to the scientific community, but also to the people impacted by the science.

**Patient or Public Contribution:**

PWLE were engaged throughout the scoping review process, including the screening phase, analysis phase, and write‐up phase.

## 1 INTRODUCTION

The past two decades have demonstrated a shift towards engaging people with lived experience (PWLE) in health research as collaborators, rather than as study participants.^1^ Often referred to as patient engagement, consumer engagement, patient and public involvement, or co‐production, engagement in research involves authentic and ongoing collaboration with PWLE across the research cycle, from conceptualization to dissemination.^2^ PWLE can be engaged on a continuum, ranging from consultation and advisory roles to equal partnerships, leadership, and decision‐making roles.^3^ Engagement in research has been framed as a way to improve research quality and relevance of study findings to the community, in addition to being an ethical imperative.^4^

Funding bodies are increasingly interested in supporting researchers who engage PWLE throughout the research process to improve the impact, quality, and relevance of the research they fund.^5^, ^6^, ^7^ For instance, institutions such as the Centre for Engagement and Dissemination in the United Kingdom,^8^ Patient‐Centered Outcomes Research Institute in the United States,^6^ and the Canadian Institutes of Health Research Strategy for Patient‐Oriented Research in Canada^2^ have set guidelines and policies around engagement in health research. As institutions continue to set national standards around engagement, efforts should be made to mitigate potential harms from tokenistic and inequitable practices.^9^ This is especially important as engagement in research is often critiqued for having limited representation of socially marginalized groups (e.g., racialization, low income) among collaborators.^10^, ^11^

The shift from passive recipients to active experts, researchers, and leaders has fueled an expanding body of democratic research in the mental health and substance use field. The moral obligation of engagement is especially relevant to this field given the historical oppression and coercive practices in psychiatry that have left patients silenced, without power, and as passive recipients of care.^12^ Grassroots movements in the late twentieth century played a key role in dismantling power dynamics and challenging current practices in psychiatry, along with advocating for the involvement of PWLE as key knowledgemakers within systems restructuring and research.^12^, ^13^ Yet, there is work to do to continue unpacking past practices, especially as ongoing progress is often overshadowed by research priorities rather than challenging power structures within institutions.^14^

There is growing evidence demonstrating the impact of engagement in health research. Previous engagement research suggests that the way impact is measured and reported is inconsistent and limited to subjective accounts of impacts.^15^, ^16^, ^17^ Evidence from reviews that assess impact varies regarding the impact on *who*, such as the impact on individuals (e.g., PWLE and researchers)^18^ or *what*, such as the impact on research design and delivery (e.g., recruitment rates in clinical trials).^19^, ^20^ Similar to how evidence‐based medicine is viewed as the gold standard in health research, current practices in health research largely focused on measuring the impact of engagement on the research process.^9^ However, especially in the mental health field, framing engagement as solely valuable to the research itself risks undermining the ethical imperative behind engagement activities.^21^

Recent reviews have described the impact of engagement in health research;^18^, ^20^, ^22^ however, it remains unclear what evidence is available regarding the impact of engagement specific to mental health and substance use research. Indeed, the assumptions underlying impact in health research may not apply to a mental health and substance use context given the abundant presence of power imbalances, the perceived vulnerability of PWLE, and the stigma around the capacity of PWLE to consult in research projects.^12^, ^13^ Therefore, this scoping review aims to map the literature on how the impact of engagement is conceived in mental health and substance use research. A scoping review is ideal for this study as the topic is an emerging field, with a wide range of designs across studies, allowing for a flexible approach—an important feature as the state of the evidence and methods is unclear.^23^ The specific objectives of this review are to map the impact of engagement on both individual experience and the research process and identify key barriers and facilitators to impactful engagement. The following research questions are identified:

1. What is known from the existing empirical literature about the impact of engagement in mental health and substance use research?
2. What are the barriers and facilitators to impactful engagement in mental health and substance use research?

## 2 METHODS

The present scoping review was guided by Arksey and O'Malley's framework^24^ and the enhanced framework by Levac and colleagues.^25^ The Preferred Reporting Items for Systematic Reviews and Meta‐Analyses Extension for Scoping Reviews checklist was followed to ensure methodological and reporting quality.^26^ This review follows the Canadian Institutes of Health Research (CIHR) definition of patient engagement in research, which is defined as ‘meaningful and active collaboration in governance, priority setting, conducting research and knowledge translation’.^2^ The term ‘patient engagement’ is only used for clarity purposes as it is commonly used in health research. In response to the expressed preferences of our lived experience panel, we refer to people with lived experience engaged in research projects as PWLE instead of the term ‘patient’—this is for clarity purposes; it should be noted that researchers can also identify as PWLE and apply their lived experience to research.^27^, ^28^

### 2.1 Patient and public involvement

The scoping review process involved PWLE during the screening phase, analysis phase, and write‐up phase. As detailed in Section 2.4, the screening phase involved two PWLE. During the analysis phase, the results were presented to the Lived Experience Advisory Group at the Centre for Complex Interventions within CAMH. Based on the feedback from the first meeting, the results were refined to replace ‘patient’ with ‘people with lived experience,’ in addition to examining terminology in the analysis. The refined results were brought back to the group in the second meeting during the write‐up phase. Lastly, multiple manuscript versions were further refined by two PWLE, who are also included as co‐authors.

### 2.2 Eligibility criteria

Eligibility criteria are shown in Table 1. The Population, Concept, and Context (PCC) framework by the Joanna Briggs Institute was used to identify relevant studies for this scoping review.^29^ For the *population*, this review includes studies that focus on PWLE of mental health or substance use challenges (any age group), and have been engaged or involved in mental health or substance use research as collaborators (e.g., advisors, co‐researchers). The *concept* of the scoping review is the impact of engagement, including reported outcomes on individual experiences and the research process. The *context* of the review is limited to engagement in mental health and substance use research in the past decade to capture the impacts related to the current state of engagement practices. Articles that look at engagement outside of a research context, for example only in the context of health care policy and governance work, were excluded as the justification, processes, and impact may differ. There were no limitations for the geographical location.

**Table 1:** Search criteria for the scoping review.

Search terms	Concept 1: Engagement	Concept 2: Mental Health	Concept 3: Impact
	Patient participation/**OR** (patient* or client* or public or ‘service user*’ or youth or consumer* or citizen*) adj2 (participat* or engag* or invol*) **OR** ‘liv* expertise’ or ‘lived experience’ or ‘peer* researcher*’ or ‘co‐researcher*’ or ‘expert* by experience*’ or ‘patient* partner*’ or ‘patient* advisor*’ or ‘co‐produc*’ or ‘co‐design’	Mental Health/**OR** (mental* or psychiatr* or psycholog*) adj2 (health* or ill* or hygiene or disorder* or distress*) **OR** (drug* or substance* or alcohol*) adj2 (abus* or addict* or depend* or misus* or use* or dependen* or disorder*)	(improv* or strength* or inform* or increase* or impact* or facilitat* or support*) adj3 (research* or method* or design or outcome* or recruit* or study or team*)
Databases	Medline (Ovid), CINAHL (EBSCO), and PsycINFO (ProQuest)		
Inclusion	(a) academic journal articles, including full‐length original research (e.g., qualitative, quantitative, mixed or multi‐methods, case studies), brief reports, or commentaries/viewpoints that provide an overview of the engagement process in a project		
	(b) focused on ‘patient engagement’ in a research context		
	(c) specific to mental health or substance use research		
	(d) published in English		
	(e) published between 2012 and 2022		
Exclusion	(a) defined ‘patient engagement’ as patient retention or engagement in clinical decisions (e.g., patient care and shared decision‐making)		
	(b) did not include a research context (e.g., studies looking at engagement in non‐research government setting)		
	(c) did not describe the impact of engagement		
	(d) focused primarily on neurological, developmental, or physical disorders		
	(e) reviews, protocols, conference abstracts		
	(f) focused only on family engagement		
	(g) commentaries/viewpoints that did not provide a description of the engagement process in a project		
Time	The scoping review was conducted in June 2022 and included studies published between the period of January 2012 and June 2022.		

*Note*: An asterisk is used to represent a ‘wildcard’ or unknown character.

### 2.3 Information sources and search strategy

The search strategy was developed in consultation with a health sciences librarian. The following electronic databases were searched in June 2022: Medline (Ovid), CINAHL (EBSCO), and PsycINFO (ProQuest). Academic articles were limited to after 2012 due to engagement in mental health and substance use research growing substantially in the past decade; it also maximized relevance to current engagement frameworks.

A full description of search terms and strategies is shown in Table 1. As ‘patient engagement’ is broadly defined, without consistent terminology, multiple search strategies were piloted to identify appropriate keywords. After an initial search was conducted to determine which terms reflect the phenomena in the research question, the following combination of search terms were used to broadly define PWLE, such as patient*, client*, public, service user*, youth, consumer*, and citizen*. To capture engagement, search terms such as engag*, participat*, and invol* were used adjacent to the term for PWLE, in addition to other terms such as co‐researcher*, co‐design*, and co‐produc*. Search terms such as mental, psychiatr*, psycholog*, substance* were used adjacent to health*, use*, disorder* to situate the search in the field of mental health and substance use. Lastly, search terms such as impact*, support*, and improv* were included to capture studies that discussed the impact. The search terms were adapted for each database concerning the proximity operators, truncations, and wildcard symbols.

### 2.4 Selecting sources of evidence

All articles identified in each database search were imported to Covidence (www.covidence.org), a systematic review software. Upon uploading the search results from each database, duplicates were removed. Titles and abstracts were screened by three reviewers (including two PWLE) for relevance to the PCC eligibility criteria. Following this step, two reviewers screened 20 of the 160 articles selected for full‐text review, yielding a *κ* value of 1. Given the perfect agreement, one reviewer screened the remaining articles to further validate eligibility and identify relevant publications from the listed sources. A total of 61 articles were deemed eligible for data extraction.

### 2.5 Data charting and analyses

Informed by Arksey and O'Malley and Levac and colleagues,^24^, ^25^ a data charting tool was developed iteratively by NYS and LDH to extract information from the 61 articles. The data charting form was created on Microsoft Excel and initially piloted on 20 articles, enabling the refinement of the charting tool before its use in this study. The tool included article characteristics, key variables relevant to the PCC criteria, and findings related to the research questions. The following data were abstracted: (1) general information (authors, title, publication year, journal, country, article type); (2) study design (population, setting, methods, objectives, whether a reporting guideline is used); (3) engagement context (level of engagement, whether sociodemographics for PWLE such as gender and race/ethnicity were reported, the term used to describe engagement, whether PWLE were included as co‐authors); (4) concepts (outcomes, focus on individual experiences or research process); and (5) key findings (impacts, facilitators, barriers). Note, data on PWLE as co‐authors were removed due to the subjectivity and uncertainty of accurately identifying their lived experience status in the authorship list. Facilitators and barriers were added as data items during the pilot data charting process due to the dominance of these factors in the selected articles and the richness of information they provided.

In addition to the data charting tool, the 61 articles were uploaded to NVivo 12 and analysed by a single coder (NYS) using the codebook approach to thematic analysis.^30^ Here, themes are conceptualized as topic summaries.^30^ An initial coding framework was developed by NYS based on the piloted data charting process. Throughout the process, additional codes and themes were developed inductively and refined through weekly meetings with two of the authors (NYS, LDH). The codes and themes were further refined through feedback from a larger research team within the same unit, of which many team members identified as having lived experience, in addition to the unit's Lived Experience Advisory Group. Lastly, using the text frequency option on NVivo 12, we explored the most commonly used terminologies for ‘engagement’ and ‘people with lived experience’. This was added at a later phase as a separate analysis from the thematic analysis and was based on the feedback from the Lived Experience Advisory Group.

### 2.6 Synthesis of results

The extracted data from Excel and NVivo were collated and summarized in a narrated format according to the key outcomes related to the research questions: impact on individuals, impact on the research process, and facilitators and barriers to impactful engagement. The evidence is also presented through tables and a diagram. The tables include the study characteristics, findings related to the facilitators and barriers, and commonly used terminologies. A figure is used to summarize the impact of engagement on PWLE, researchers, study participants, and the research process.

## 3 RESULTS

**Figure 1:**
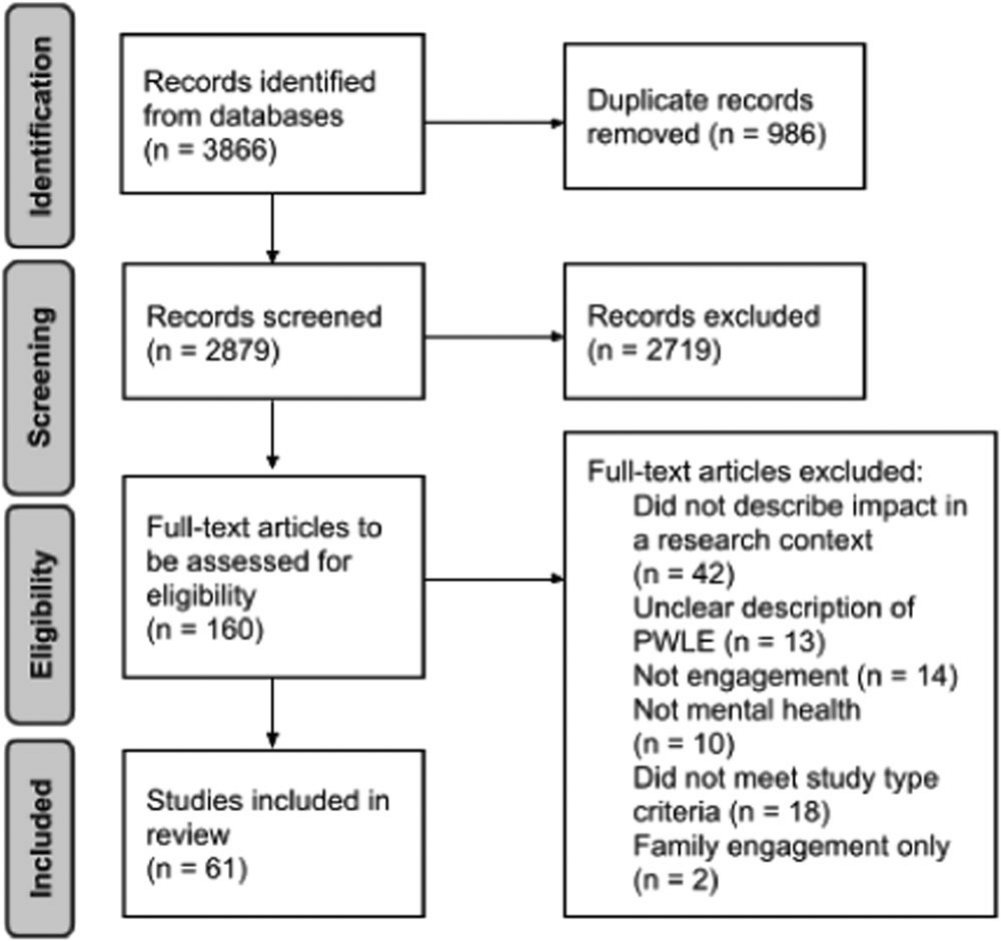
PRISMA‐ScR flow diagram of article selection. PRISMA‐ScR, Preferred Reporting Items for Systematic Reviews and Meta‐Analyses Extension for Scoping Reviews.

**Figure 2:**
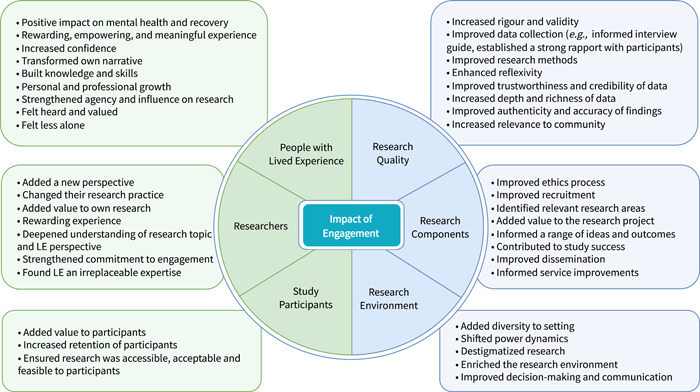
The impact of engagement on individuals and the research process in mental health and substance use research. LE, Lived experience.

The search strategy yielded 2879 citations after removing 986 duplicates. After reviewing 2879 titles and abstracts, followed by 160 full‐text records, 61 articles were included in the scoping review (Figure 1). The majority of studies were published in 2019–2022 and came from the United Kingdom, Australia, or Canada (Table 2). The 61 articles consist of 21 reflection/description of process papers, 20 qualitative studies, 5 quantitative studies, 4 viewpoints/commentaries, 3 case studies, and 1 priority‐setting paper. Key topics include (a) the impact of engagement on individual experiences; (b) the impact of engagement on the research process; and (c) facilitators and barriers to impactful engagement. All 61 articles described the impact of engagement on either individual experiences or the research process (Figure 2).

**Table 2:** Study characteristics (total = 61 studies).

Characteristic	Count
*Study type*	
Reflection or description of the process	21
Qualitative	20
Mixed or multi‐method	7
Quantitative	5
Viewpoint or commentary	4
Case study	3
Priority setting	1
*Country*	
Canada	12
United States	7
United Kingdom	23
Australia	13
New Zealand	1
Ireland	1
Germany	1
Norway	1
Sweden	1
*Year of publication*	
2019–2022	36
2015–2018	18
2012–2014	7
*Guidelines followed*	
Followed GRIPP or GRIPP2	8
Did not follow GRIPP or GRIPP2	53

Abbreviations: GRIPP, Guidance for Reporting Involvement of Patients and the Public.

### 3.1 Impact of engagement on individual experiences (*N* = 48)

Perceived impacts related to individual experiences were described in 48 articles (Figure 2). Here, 35 articles described the impact of PWLE, 26 articles described the impact on researchers, and 11 articles described the impact on study participants.

#### 3.1.1 Impact on people with lived experience (*N* = 35)

The impact of engagement on PWLE was described in 35 articles. Engagement activities were seen as providing positive opportunities for PWLE, including personal and professional growth,^31^, ^32^, ^33^, ^34^, ^35^, ^36^, ^37^, ^38^, ^39^, ^40^, ^41^ in addition to building knowledge and skills.^32^, ^33^, ^34^, ^35^, ^37^, ^38^, ^39^, ^40^, ^42^, ^43^, ^44^, ^45^, ^46^, ^47^, ^48^, ^49^, ^50^, ^51^ Many studies noted that PWLE found engagement to be a meaningful, empowering, and rewarding experience.^32^, ^34^, ^42^, ^43^, ^52^, ^53^ Moreover, PWLE described the impact of engagement on the self, such as feeling heard and valued,^31^, ^32^, ^35^, ^36^, ^38^, ^43^, ^45^, ^49^, ^50^, ^51^, ^54^, ^55^, ^56^, ^57^, ^58^, ^59^ building confidence,^32^, ^34^, ^37^, ^38^, ^39^, ^41^, ^42^, ^54^, ^60^, ^61^, ^62^, ^63^ feeling less alone in their experiences,^38^, ^63^ transforming their narrative (e.g., new self‐understanding),^31^, ^34^, ^39^, ^41^, ^43^, ^49^, ^50^, ^54^ and having a positive impact on their mental health and recovery.^36^, ^39^, ^41^, ^42^, ^52^, ^65^ Lastly, PWLE reported feeling a sense of strengthened agency and control, including their ability to influence and change research.^31^, ^32^, ^33^, ^41^, ^46^, ^66^, ^67^

#### 3.1.2 Impact on researchers (*N* = 26)

Despite 26 articles reporting on the impact of engagement on researchers, the findings are less extensive compared to PWLE. Several studies noted that researchers found engagement to be valuable and rewarding,^35^, ^37^, ^42^, ^44^, ^45^, ^65^, ^67^, ^68^, ^69^ giving them a deeper understanding of the research topic and lived experience perspectives.^43^, ^44^, ^47^, ^54^, ^56^, ^70^ Researchers also found that engagement added value to their research,^35^, ^44^, ^47^, ^62^, ^71^, ^72^, ^73^, ^74^ brought a new perspective to the research project,^38^, ^44^, ^49^, ^65^, ^69^ and was a valuable means of bringing about change in research.^75^ In three studies, researchers reported finding lived experience as an irreplaceable expertise.^44^, ^51^, ^69^ Engagement resulted in changes to their research practice,^37^, ^39^, ^47^, ^59^, ^60^, ^65^, ^76^ in addition to strengthening their commitment to engagement going forward.^37^, ^47^, ^67^

#### 3.1.3 Impact on study participants (*N* = 11)

Although the evidence is limited, some studies reported that engagement had a positive impact on the experiences of participants in a research project. Including PWLE in the research process was seen as an added value to participants,^32^, ^39^, ^68^ was considered to have made a positive difference to participants,^63^, ^77^ and was found to create a safe space for participants.^57^, ^78^ Moreover, two studies noted that engagement increased the retention of study participants.^44^, ^77^ Overall, engagement helped ensure that the research was accessible, acceptable, and relevant to participants^50^, ^65^, ^77^ and that study materials were appropriate for participants.^51^, ^59^

### 3.2 Impact on the research process (*N* = 50)

Fifty studies described the impact of engagement on the research process (Figure 2). The impact was threefold: engagement positively influenced research quality, research components, and the research environment.

#### 3.2.1 Research quality (*N* = 42)

Forty‐two studies reported that engagement improved the quality and rigour of the research.^42^, ^44^, ^57^, ^66^, ^69^, ^72^, ^77^, ^79^, ^80^ Many studies reported perceived improvements to the data and findings, including improvements to data interpretation and analysis,^32^, ^37^, ^42^, ^70^, ^80^ reflexivity,^69^, ^81^ authenticity and accuracy of findings,^38^, ^57^, ^82^ trustworthiness and credibility of data,^65^, ^69^, ^79^, ^83^ depth and richness of data,^32^, ^36^, ^44^, ^51^, ^56^, ^57^, ^60^, ^63^, ^66^, ^68^, ^69^, ^80^, ^81^, ^84^ and validity.^36^, ^37^, ^57^, ^80^, ^85^, ^86^ Engagement also strengthened study design and methods.^32^, ^34^, ^39^, ^43^, ^57^, ^66^, ^67^, ^68^, ^71^, ^77^, ^83^, ^87^ For example, this included improvements to data collection for qualitative studies, such as enhanced rapport with participants^36^, ^37^, ^56^, ^63^, ^68^, ^69^ and improved interview guides.^32^, ^37^, ^44^, ^48^, ^51^, ^56^, ^68^, ^80^ Although only reported in a limited number of quantitative studies, authors considered that engagement can lead to the development of a clinically appropriate psychometric assessment,^88^ inform the selection of quantitative measures and/or analysis plan for trials,^49^, ^67^, ^87^ and inform eligibility criteria for trials.^87^ Most importantly, engagement was seen to increase relevance to the community and service users.^35^, ^36^, ^37^, ^45^, ^47^, ^48^, ^50^, ^51^, ^56^, ^57^, ^58^, ^59^, ^65^, ^68^, ^69^, ^70^, ^77^, ^80^, ^82^, ^85^, ^86^, ^87^, ^88^, ^89^

#### 3.2.2 Research components (*N* = 32)

PWLE was considered to improve research components by informing a range of ideas and outcomes;^32^, ^34^, ^43^, ^66^, ^67^, ^68^, ^71^, ^77^, ^80^ this included defining and refining research questions,^42^, ^43^, ^51^, ^68^, ^77^ identifying relevant research areas,^50^, ^55^, ^66^, ^68^ and improving the ethics review process by further highlighting ethical issues that may have otherwise been overlooked.^35^, ^44^ Several studies reported that engagement increased the recruitment of study participants.^32^, ^35^, ^42^, ^44^, ^57^, ^69^, ^72^, ^77^, ^80^ However, one study reported no impact on trial recruitment.^90^ Engagement was reported to inform and improve knowledge dissemination activities^32^, ^37^, ^48^, ^51^, ^60^, ^63^, ^68^, ^80^ and service design.^67^, ^69^, ^88^ Overall, engagement was seen as an added value to the research project^33^, ^34^, ^41^, ^43^, ^58^, ^59^, ^65^, ^67^, ^69^, ^71^, ^83^, ^89^ and was considered to contribute to study success.^33^, ^37^, ^44^, ^61^, ^67^, ^88^

#### 3.2.3 Research environment (*N* = 20)

Engagement was considered to impact both the research team and the broader research environment. In terms of the team environment, PWLE improved decision‐making and communication,^43^, ^50^, ^55^, ^67^, ^83^ fostered reciprocal learning between PWLE and researchers,^44^, ^47^, ^49^, ^57^, ^58^, ^61^, ^89^ enabled a positive change in the organizational culture,^56^, ^76^ and added diverse perspectives to the research teams.^35^, ^44^, ^69^ In terms of the broader environment, PLWE engagement challenged stigma^36^, ^40^, ^43^, ^70^ and shifted power dynamics.^54^, ^89^

### 3.3 Facilitators and barriers to impactful engagement

The majority (*N* = 51) of the studies identified facilitators and barriers to impactful engagement in mental health and substance use research (Table 3). The studies were categorized into four levels of barriers and facilitators: lived experience level, researcher level, team level, and institutional level.

**Table 3:** Barriers and facilitators to impactful engagement in mental health research.

	Barriers to impactful engagement	Facilitators to impactful engagement
*Lived experience level*	
Intrapersonal factors	Feeling anxious or nervous;^39^, ^40^, ^48^, ^59^ feeling isolated from other community partners;^60^ feeling disconnected, unsupported, or disengaged;^34^, ^36^, ^49^, ^50^, ^67^, ^80^ skepticism, mistrust, or perceived risks;^49^, ^57^, ^60^, ^62^ negative experiences;^50^ attendance issues;^32^, ^35^ varying levels of interest and availability^34^, ^40^, ^43^	Feeling accepted and valued;^32^, ^34^, ^35^, ^37^, ^38^, ^41^, ^48^, ^51^, ^56^, ^59^, ^65^, ^80^ trust;^35^, ^49^, ^51^, ^58^, ^60^ self‐awareness;^42^ seeing their feedback incorporated^32^, ^36^, ^37^, ^38^, ^45^, ^87^
Roles and responsibilities	Having their other identities ignored;^49^ given limited information;^48^ not compensated;^50^ learning curve;^67^	Contributions are formally recognized;^41^, ^51^, ^61^, ^80^ continuity with roles;^32^, ^37^, ^57^, ^67^, ^80^ given time to contribute;^32^, ^80^ having supports and resources available;^37^, ^49^, ^50^, ^55^, ^59^, ^61^, ^65^, ^76^, ^80^ included in consensus‐building or decision‐making;^37^, ^49^, ^53^, ^57^, ^63^, ^70^ co‐chairing meetings;^49^, ^60^, ^86^ expertise in addition to lived experience;^46^, ^49^, ^58^
Logistical	Technological barriers;^32^ language barriers;^34^ travel and geographical barriers;^32^, ^34^, ^57^, ^76^	Fair compensation;^39^, ^40^, ^45^, ^48^, ^50^, ^53^, ^57^, ^58^, ^59^, ^61^, ^80^ training and/or mentorship^32^, ^33^, ^35^, ^39^, ^42^, ^44^, ^50^, ^56^, ^57^, ^59^, ^61^, ^62^, ^65^, ^68^, ^71^, ^80^, ^84^, ^87^, ^91^
*Researcher level*		
Knowledge		
Attitudes and perceptions	Limited awareness of engagement opportunities;^65^ limited understanding of patient experiences;^75^ not knowing how to properly engage;^91^ pushback from researchers;^51^, ^61^, ^72^ only participating in engagement activities because it is required;^77^ valuing institutional knowledge over lived experience;^47^, ^50^, ^61^, ^65^, ^75^ researcher identity^66^; paternalistic attitudes (e.g., patronizing)^75^	Listening and open to feedback;^41^, ^65^ recognizing power differences;^65^, ^80^, ^85^ advocating for engagement;^45^, ^51^, ^58^, ^75^ valuing lived experience as an expertise;^38^, ^45^, ^49^, ^61^ perceived support from colleagues^72^
Logistical		Engagement training and/or mentorship for researchers;^40^, ^47^, ^72^, ^77^, ^91^
*Team level*		
Communication	Poor communication;^34^, ^40^, ^46^, ^48^, ^50^ use of jargon;^39^, ^49^, ^57^, ^61^, ^65^, ^80^ not integrating feedback from PWLE^65^, ^80^	Pre‐ and de‐briefs;^37^, ^39^, ^49^, ^55^, ^57^, ^61^, ^67^, ^78^ listening to each other;^37^, ^38^, ^41^, ^45^ clearly defined roles;^45^, ^48^, ^50^, ^57^, ^61^, ^80^ honest and open conversations;^37^, ^49^, ^55^, ^80^ transparent and clear communication;^49^, ^57^, ^60^, ^61^, ^80^, ^87^ plain language;^59^, ^67^, ^79^, ^85^ understanding different preferences;^49^, ^59^, ^80^ strong support and values set as early as possible;^43^, ^62^, ^67^ reciprocity between researchers and patients.^31^, ^33^
Team dynamics/interactions	Differing or conflicting views;^36^, ^37^, ^49^, ^50^, ^65^, ^67^, ^76^, ^85^ tokenism;^33^, ^36^, ^42^, ^53^, ^61^, ^65^, ^67^, ^71^, ^72^, ^77^, ^80^, ^85^ stigma and/or prejudice^36^, ^40^, ^42^, ^47^, ^49^, ^50^, ^51^, ^75^, ^76^	Building trust;^37^, ^49^, ^57^, ^70^, ^85^ established relationships or rapport early on;^37^, ^40^, ^46^, ^58^, ^67^ inclusive, safe, and non‐judgmental team environment;^37^, ^38^, ^42^, ^45^, ^49^, ^58^, ^59^, ^61^, ^64^, ^67^, ^85^ investing in team relationships;^37^, ^38^, ^49^, ^56^, ^57^, ^58^, ^60^, ^70^, ^76^, ^85^ reciprocity and mutual learning;^38^, ^41^, ^49^, ^58^, ^61^, ^76^, ^85^, ^89^ shared values;^63^ supportive and respectful team;^34^, ^35^, ^41^, ^42^, ^43^, ^49^, ^55^, ^56^, ^57^, ^58^, ^59^, ^61^, ^63^, ^67^, ^71^, ^72^, ^77^, ^84^, ^85^ power‐sharing^34^, ^36^, ^37^, ^38^, ^46^, ^47^, ^49^, ^53^, ^56^, ^57^, ^58^, ^63^, ^67^, ^70^, ^80^, ^87^
Planning and implementation	Lack of diversity/representativeness;^43^, ^50^, ^67^, ^80^ missing or limited engagement in early stages;^50^, ^65^, ^87^ transactional/superficial involvement;^36^, ^80^ disorganized;^50^ lack of continuity with roles^72^, ^80^	Anti‐oppressive and/or trauma‐informed lens;^36^, ^53^, ^58^, ^61^, ^85^ strong commitment to engagement;^32^, ^48^, ^72^ engagement at early stages;^40^, ^50^, ^59^, ^76^, ^77^, ^80^, ^84^, ^85^, ^87^, ^89^ engagement throughout the research process;^40^, ^43^, ^77^, ^80^, ^84^ diverse representation of lived experiences;^37^, ^53^, ^55^, ^56^, ^80^ flexibility throughout the research process;^32^, ^37^, ^40^, ^45^, ^46^, ^47^, ^49^, ^57^, ^59^, ^60^, ^61^, ^70^, ^76^, ^77^, ^80^, ^87^ providing a range of opportunities;^35^, ^55^ having an engagement coordinator;^50^, ^60^, ^67^, ^72^, ^80^ well‐planned engagement;^32^, ^80^ ongoing reflective practice^57^, ^87^
*Institutional level*		
Resources	Time constraints;^37^, ^42^, ^53^, ^62^, ^65^, ^66^, ^67^, ^71^, ^72^, ^76^, ^80^, ^87^, ^92^ limited funding and financial resources;^32^, ^37^, ^48^, ^57^, ^62^, ^65^, ^66^, ^67^, ^72^, ^75^, ^77^, ^80^, ^91^ reliance on one organization^43^	External partnerships;^35^, ^43^, ^48^, ^50^, ^54^, ^55^, ^57^ networks and more resources available;^65^, ^91^ establishing a lived‐experience researcher group;^73^, ^74^ support from organization;^62^, ^65^, ^92^ support from funders;^62^, ^77^, ^91^ incentives for engagement^77^
Institutional structures and culture	Competitive nature of research environment;^42^, ^59^, ^66^, ^67^, ^71^, ^72^, ^76^, ^77^ research culture;^49^, ^51^, ^53^, ^76^, ^77^ resistance to change;^47^, ^67^, ^75^, ^91^ hierarchies within research;^49^, ^51^, ^75^ bureaucratic requirements;^47^, ^57^, ^76^ ethics board;^47^, ^59^, ^62^, ^75^, ^76^, ^77^ power differences;^36^, ^40^, ^43^, ^47^, ^49^, ^51^, ^57^, ^60^, ^72^, ^75^, ^91^ ‘us’ versus ‘them’ culture (e.g., having their lived experience ignored, not disclosing their lived experience);^49^, ^58^ lack of community accountability for researchers^36^	Organization's readiness for change;^56^ lived experience representation at the leadership level;^32^, ^53^ institutional requirements for engagement;^73^ expectations set by funders or institutions for high levels of engagement;^62^, ^70^, ^72^, ^74^, ^91^ flexibility within hospital structures;^32^, ^33^

#### 3.3.1 Lived experience level (*N* = 35 studies)

The literature related to facilitators and barriers at the level of PWLE included personal factors that influence engagement, such as emotions and perceptions, roles and responsibilities, and others. Most common facilitators included training and/or mentorship,^32^, ^33^, ^35^, ^39^, ^42^, ^44^, ^50^, ^56^, ^57^, ^59^, ^61^, ^62^, ^65^, ^68^, ^71^, ^80^, ^84^, ^87^, ^91^ feeling accepted and valued,^32^, ^34^, ^35^, ^37^, ^38^, ^41^, ^48^, ^51^, ^56^, ^59^, ^65^, ^80^ having supports and resources available,^37^, ^49^, ^50^, ^55^, ^59^, ^61^, ^65^, ^76^, ^80^ and fair compensation.^39^, ^40^, ^45^, ^48^, ^50^, ^53^, ^57^, ^58^, ^59^, ^61^, ^80^ Common barriers included PWLE feeling disconnected, unsupported, or disengaged,^34^, ^36^, ^49^, ^50^, ^67^, ^80^ in addition to skepticism, mistrust, or perceived risks.^49^, ^57^, ^60^, ^62^

#### 3.3.2 Researcher level (*N* = 18 studies)

Researcher‐level facilitators and barriers were less frequently reported compared to other levels and largely related to researchers' knowledge, attitudes, and perceptions regarding engagement. Some studies noted facilitators such as recognizing power differences,^65^, ^80^, ^85^ advocating for engagement,^45^, ^51^, ^58^, ^75^ and valuing lived experience as expertise.^38^, ^45^, ^49^, ^61^ Engagement training and mentorship was also seen as an important facilitator for researchers.^40^, ^47^, ^72^, ^77^, ^91^ Common barriers included researchers valuing institutional knowledge over lived experience,^47^, ^50^, ^61^, ^65^, ^75^ and pushback from researchers.^51^, ^61^, ^72^

#### 3.3.3 Team level (*N* = 45 studies)

Team‐level facilitators and barriers are the most frequently reported, described across 45 studies. Facilitators and barriers related to planning and implementation, communication, and team interactions. Critical facilitators for the planning and implementation of successful engagement include engaging PWLE at early stages in the research process^40^, ^50^, ^59^, ^76^, ^77^, ^80^, ^84^, ^85^, ^87^, ^89^ and flexibility throughout the research process.^32^, ^37^, ^40^, ^45^, ^46^, ^47^, ^49^, ^57^, ^59^, ^60^, ^61^, ^70^, ^76^, ^77^, ^80^, ^87^ Barriers include a lack of diversity among PWLE^50^, ^65^, ^87^ and limited engagement in early stages.^43^, ^50^, ^67^, ^80^ Common communication facilitators include holding pre‐ and de‐briefs,^37^, ^39^, ^49^, ^55^, ^57^, ^61^, ^67^, ^78^ ensuring transparent and clear communication,^49^, ^57^, ^60^, ^61^, ^80^, ^87^ and clearly defining roles.^45^, ^48^, ^50^, ^57^, ^61^, ^80^ Barriers at the team level include the use of jargon among team members.^38^, ^49^, ^57^, ^61^, ^65^, ^80^ Several studies referenced facilitators regarding team interactions, such as supportive and respectful teams.^34^, ^35^, ^41^, ^42^, ^43^, ^49^, ^55^, ^56^, ^57^, ^58^, ^59^, ^61^, ^63^, ^67^, ^71^, ^72^, ^77^, ^84^, ^85^ The current literature also suggests the importance of inclusive, safe, and non‐judgmental team environments for successful engagement activities.^37^, ^38^, ^42^, ^43^, ^49^, ^58^, ^59^, ^61^, ^64^, ^67^, ^85^ However, tokenism^33^, ^36^, ^42^, ^53^, ^61^, ^65^, ^67^, ^71^, ^72^, ^77^, ^80^, ^85^ and conflicting views^36^, ^37^, ^49^, ^50^, ^65^, ^67^, ^76^, ^85^ are frequent barriers to the successful engagement of PWLE.

#### 3.3.4 Institutional level (*N* = 37 studies)

Barriers and facilitators to engagement at the institutional level are described across 37 studies. These relate to the institution's culture and structures, in addition to the resources provided by institutions (e.g., resources from organizations, and funding bodies). For the institution's culture and structure, the expectations set by institutions for high levels of engagement^62^, ^70^, ^72^, ^74^, ^91^ facilitated impactful engagement. However, commonly cited barriers included power differences,^36^, ^40^, ^43^, ^47^, ^49^, ^51^, ^57^, ^60^, ^72^, ^75^, ^91^ the competitive nature of the research environment (e.g., fast‐paced, heavy focus on outputs),^42^, ^59^, ^66^, ^67^, ^71^, ^72^, ^76^, ^77^ and the research culture itself.^49^, ^51^, ^53^, ^76^, ^77^ In terms of resources, external partnerships^35^, ^43^, ^48^, ^50^, ^54^, ^55^, ^57^ and support from funding bodies^62^, ^77^, ^91^ were seen as critical facilitators, while time constraints^37^, ^42^, ^53^, ^62^, ^65^, ^66^, ^67^, ^71^, ^72^, ^76^, ^80^, ^87^, ^92^ and limited funding^32^, ^37^, ^48^, ^57^, ^62^, ^65^, ^66^, ^67^, ^72^, ^75^, ^77^, ^80^, ^91^ were major barriers.

### 3.4 Commonly used terminologies

The most commonly used terminologies for engagement and PWLE are shown in Table 4. Out of 61 studies, commonly used terminology for engagement across the included studies was ‘participatory research’ (31.1%), ‘patient and public involvement’ (29.5%), ‘coproduction’ (27.9%), and ‘service user involvement’ (23.0%). Commonly used terminology for people with lived experience included ‘patient’ (59.0%), ‘service user’ (52.5%), and ‘consumer’ (34.4%).

**Table 4:** Most commonly used terminology related to engagement across studies (*N* = 61).

	Frequency^b^	%
*Most commonly used term for “engagement” in each study^a^*		
Participatory research	19	31.1
Co‐production	17	27.9
Patient and public involvement	18	29.5
Service user involvement	14	23.0
Consumer involvement	8	13.1
Co‐design	8	13.1
Patient engagement	7	11.5
Consumer research	7	11.5
Youth engagement	4	6.6
Youth participation	4	6.6
Youth involvement	4	6.6
Lived experience involvement	3	4.9
Lived experience research	3	4.9
Involvement of people with lived experience	2	3.3
Peer co‐facilitation	1	1.6
Expert by experience involvement	1	1.6
*Most commonly used term for “people with lived experience” in each study*		
Patient	36	59.0
Service user	32	52.5
Consumer	21	34.4
Co‐researcher	19	31.1
Peer	15	24.6
Youth	16	26.2
People with lived experience	13	21.3
Young people	12	19.7
Experiential expert	9	14.8
Consumer researcher	8	13.1
Service user researcher	8	13.1
Peer researcher	4	6.6
Individuals with lived experience	4	6.6
Expert by experience	3	4.9
Peer worker	2	3.3
Community partner	1	1.6
Patient researcher	1	1.6
Lived experience researcher	1	1.6
Individual with lived expertise	1	1.6

*Note*: (a) Excludes reference list. (b) Frequency refers to occurrence of term across the included studies.

## 4 DISCUSSION

This scoping review identified 61 articles that discuss the impact of engagement in mental health and substance use research between 2012 and 2022 and provides an overall picture of the available evidence on engagement in the mental health and substance use field. Overall, engagement was considered to have a positive impact on individual experiences and the research process. Most studies focused on subjective accounts when reporting on impact; this included a range of positive impacts on PWLE, researchers, and study participants. Engagement activities were commonly reported to improve facets of the research process, such as research quality, research components, and the research environment. As the review additionally synthesizes facilitators and barriers to engagement at the lived experience, researcher, team, and institutional levels, it can serve as a foundation for future research aiming to engage PWLE in mental health and substance use research.

### 4.1 Epistemic versus ethical justification for impact

Our findings indicate similarities between mental health, substance use, and other health research fields regarding the perceived impacts on the research process. For instance, reviews in health research have reported the positive impact of engagement on research design, study recruitment,^93^ and data collection.^6^, ^93^, ^94^ These findings demonstrate an emphasis on epistemic benefits, such as the 3R's of research—rigour (e.g., rigour related to study design), relevance (e.g., relevance to population needs), and reach (e.g., dissemination and knowledge translation).^95^ Note, engagement in mental health research is critiqued for overemphasizing epistemic benefits rather than ethical ones.^21^ If research teams focus solely on impacts related to epistemic benefits while neglecting ethical imperatives, the justification for engagement risks being treated as a means to an end of achieving better research, rather than being an intrinsically good and democratic process.^96^

Much like the studies included in our review, the broader health literature focuses on process indicators and the perceived impact of engagement rather than empirical outcomes.^6^, ^20^ For example, a recent systematic review by Wiles et al.^20^ found that measures for the effect of engagement in randomized controlled trials are limited to process indicators such as recruitment. Moreover, Forsythe et al.^6^ note in their review that the subjective nature of study descriptions, as well as the lack of experimental studies, make it difficult to measure the magnitude of engagement impacts. Reviews have additionally highlighted the need for quantitative and experimental studies that measure empirical outcomes of engagement, including the costs and benefits of engagement.^97^, ^98^ However, others argue that quantitative approaches are less useful for evaluating the impact of engagement given its complex and context‐dependent nature.^94^

### 4.2 Evidence‐based medicine and power

The understanding of impact within academic institutions remains heavily influenced by evidence‐based medicine, which at times can be counterintuitive to engagement as it undervalues the needs of PWLE.^99^ Traditionally, evidence‐based medicine has been considered to devalue lived‐experience knowledge by perpetuating evidence hierarchies and failing to address power imbalances (especially apparent in the mental health field).^100^ Several barriers in our review reflect the prioritization of evidence‐based medicine over lived‐experience knowledge. Barriers such as valuing institutional knowledge over lived experience, hierarchies within research, and power imbalances retain a hierarchical view of the evidence, and ultimately, limited engagement impacts. Reflecting on our findings, recognizing power imbalances and the importance of power‐sharing should be a priority for engagement activities in mental health and substance use research.^21^

Evidence related to the impact of engagement on power differences is rarely cited in health research.^9^ However, this is less of the case for mental health and substance use research. In our review, several articles reported power‐related impacts on the research environment (e.g., challenged stigma, shifted power dynamics) and on PWLE (e.g., empowerment, strengthened agency, and control). While these findings suggest a shift away from epistemic justifications for engagement, whether and how power shifts occur across socially marginalized groups in mental health and substance use research remains unclear. For instance, applying an anti‐oppressive lens to engagement activities was only mentioned in a few studies.^36^, ^53^, ^85^ Additionally, the impact of institutional changes on diffusing power imbalances in mental health and substance use research should be further explored. This could include processes such as embedding PWLE in research leadership roles such as voting members on executive committees (as demonstrated in the ACCESS Open Minds network^53^) and as co‐chairs (as illustrated in the PARTNERS2 research program^49^ and DeStress study^60^).

### 4.3 Individual experiences

Discussion of the impact on individual experiences is emerging, yet remains limited in mental health and substance use research. Evidence from this review indicates the perceived value of engagement to PWLE, researchers, and study participants. Almost a decade ago, a review by Brett et al.^18^ examined the impact of engagement in health research on individuals, including PWLE and researchers. Reflecting on the present review, Brett et al.^18^ identified empowerment and skill‐building as the impact on PWLE, and a deepened understanding of their research for researchers. Other studies, such as one by Staley et al.,^101^ discuss how greater focus is needed on how engagement impacts researchers. Our review revealed limited evidence on the impact of engagement on researchers compared to PWLE—this remains a major gap in mental health and substance use research. Our findings also reveal a paucity of research on facilitators to engagement at the researcher level. These gaps should be further investigated as the positive impact on researchers could potentially shift attitudes toward engagement within institutions, lifting a barrier to engagement.^91^

### 4.4 Inconsistent reporting

At the same time as the debates on the justification for evaluating impact, researchers have also highlighted inconsistent reporting of engagement in health research.^102^ Our findings demonstrate a similar case for mental health and substance use research. Across the included studies, there were inconsistencies with terminologies used for engagement, and considerable variation in the details provided on engagement activities, characteristics of PWLE involved, and impact on the research. Reporting guidelines such as the GRIPP2 checklist can promote transparency and consistency when reporting on the impact of engagement in a study.^16^, ^102^ However, the GRIPP2 checklist is not specific to mental health and substance use research, which may explain why it was followed in only 8 of the 61 articles included in this review. The use of reporting guidelines for engagement in mental health and substance use research should be further explored, including the development of a guideline specifically tailored to the unique characteristics and challenges of this field. Without consistent reporting, a fulsome understanding of the impact of engagement in mental health and substance use research—whether on individuals or the research process—will remain unclear.

### 4.5 Strengths and limitations

The strengths of this study include the use of multiple databases, a comprehensive search strategy, and thematic analysis, in addition to engaging with PWLE throughout the scoping review process. However, several limitations warrant consideration. First, the review was limited to academic journal articles; it is possible that the grey literature could include facets missing in the academic press related to impact. Future reviews may benefit from expanding the search strategy to include the grey literature. Second, the articles came from high‐income countries and were only in English; therefore, they may not be generalizable to other settings. Third, the review may have missed relevant articles given the wide range of terminology related to engagement in mental health and substance use research. Similarly, articles with PWLE as researchers were not explored (e.g., survivor‐led research), which is an important topic for future research. Fourth, the inconsistent and largely subjective impacts reported across the literature may have influenced the study findings. Future research may benefit from standardized reporting of engagement impacts for reproducibility; however, this should not overshadow the ethical imperatives for engagement. Lastly, no assessment of the risk of bias or critical appraisal was conducted for this review, although this is not required for scoping reviews.^26^

## 5 CONCLUSION

This scoping review presents a comprehensive overview of the current literature on engagement impacts in mental health and substance use research. Additionally, facilitators and barriers to impactful engagement were identified at the lived experience, researcher, team, and institutional levels, which can be leveraged to improve engagement practices. Engaging PWLE throughout the research cycle—from consultation to co‐creation—was perceived as having a positive impact on both the research process and individual experiences. Future research is needed to bring consistency to engagement, leverage the facilitators to engagement, and address the barriers such as power differences. This would in turn generate research findings that have value to the scientific community, but also to the people impacted by the science.

## AUTHOR CONTRIBUTIONS

Natasha Y. Sheikhan was responsible for leading all stages of the scoping review. Natasha Y. Sheikhan and Lisa D. Hawke contributed to the study conception and design. Material preparation, data collection and analysis were performed by Natasha Y. Sheikhan, Lisa D. Hawke, and Shelby McKee. All authors contributed to the interpretation of emerging findings through several rounds of feedback on the draft manuscript, which was drafted by Natasha Y. Sheikhan. All authors read and approved the final manuscript.

## CONFLICT OF INTEREST STATEMENT

The authors declare no conflict of interest.

## ETHICS STATEMENT

Institutional ethics approval was not required for this scoping review as it retrieved and synthesized data from already published studies.

## Data Availability

Data that support the findings of this study are available from the corresponding author upon reasonable request.
